# Dual-Isotope Scintigraphy for Gastrointestinal Transit in Duodenal Switch: An Explorative Clinical Study

**DOI:** 10.1007/s11695-025-08146-3

**Published:** 2025-08-08

**Authors:** Filip Möller, Martin Skogar, Lucyna Neuger, Charles Widström, Magnus Sundbom

**Affiliations:** 1 Department of Surgical Sciences, Uppsala University, Uppsala, Sweden; 2 Uppsala University Hospital, Uppsala, Sweden

## Abstract

**Introduction:**

Biliopancreatic diversion with duodenal switch (BPD/DS) is a highly effective metabolic bariatric procedure. However, the decreased gastric volume and exclusion of small bowel to reduce uptake of fat-soluble nutrients can induce gastrointestinal side effects. The aim of this study was to assess gastrointestinal transit times for bile and food using a novel dual-isotope scintigraphy method and explore associations with gastrointestinal symptoms.

**Methods:**

This is an explorative single-center clinical study in 10 patients after primary BPD/DS; 99mTc Bromo-biliaron-labeled bile acid analogue was injected intravenously, 111In-DTPA-labeled omelet was ingested orally, and scintigraphic images were taken every 10 min for 180 min. Gastrointestinal symptoms were scored using the fecal incontinence quality of life (FIQL) questionnaire, and the patients were stratified by stool frequency (≥ 3 stools/day).

**Results:**

All scintigraphic examinations gave interpretable data. FIQL scores of 3.2–3.6 out of 5 demonstrated a clear quality of life impact. Gastric emptying half-time was 40 min. Median time to the enteroenterostomy was 70 min (IQR 55–138) for food and 105 min (IQR 38–148) for bile. In patients with ≥ 3 stools/day, food reached the enteroenterostomy faster than bile (70 vs 115 min), while the opposite was seen in the remaining patients (110 vs 75 min). However, no statistically significant associations between stool frequency and transit times were identified.

**Conclusion:**

Dual-isotope scintigraphy enables assessment of gastric and small bowel transit after BPD/DS. Although transit times were variable, we believe that the novel imaging technique can add valuable information in patients with severe gastrointestinal symptoms.

## Introduction

Metabolic bariatric surgery (MBS) improves obesity-related comorbidities and quality of life as a result of significant and lasting weight loss [1–3]. In patients with high body mass index (BMI) > 50 kg/m^2^, the most common MBS-procedures, Roux-en-Y gastric bypass (RYGB) and sleeve gastrectomy (SG), may lead to insufficient weight loss [4, 5], which is why many centers offer these patients a more powerful procedure, biliopancreatic diversion with duodenal switch (BPD/DS). BPD/DS combines decreased gastric volume [6], hormonal changes [7–9], and exclusion of small bowel to reduce uptake of fat-soluble nutrients [6].

These extensive anatomical changes can lead to malnutrition and gastrointestinal side effects such as diarrhea and foul-smelling flatus [10, 11]. The exact mechanism behind the adverse effects is not fully understood. The sleeve gastrectomy component reduces gastric size and alters gastric emptying [12]. The exclusion of the duodenum may impair the secretion of bile and pancreatic enzymes, leading to pancreatic insufficiency, which is seen among other patients after gastric or duodenal operations [13, 14]. A mismatch in the passage of bile and ingested food to the common channel may be an additional explanation to some gastrointestinal symptoms many patients report after BPD/DS.

The aim of this study was to assess transit times for bile and food through the gastrointestinal system in BPD/DS patients by using a novel dual-isotope scintigraphy. More specifically, assessments are of gastric emptying times and transit times to the enteroenterostomy and exploring possible correlations to gastrointestinal adverse symptoms.

## Material and Methods

### Patient Recruitment

Patients from our local MBS-registry having had primary BPD/DS more than 2 years earlier were assessed and contacted by mail for inclusion in the study. Exclusion criteria were pregnancy, current breast-feeding, multiple postoperative surgical operations, previous cholecystectomy, and chronic kidney failure (eGFR < 60). All had a classic BPD/DS, consisting of a 36-Fr sleeve gastrectomy and a 150-cm alimentary limb emptying through a side-to-side enteroenterostomy (EE) into a 100-cm common channel, while the remaining small bowel formed the biliary limb.

### Gastrointestinal Symptoms and Biochemical Profile

Patients were asked to rate their general health, gastrointestinal symptoms (stool frequency, abdominal pain, flatulence, and need for alteration of diet), and to fill out the validated fecal incontinence quality of life (FIQL) questionnaire [15]. FIQL reflects impact on lifestyle, coping, depression, and embarrassment (1–5, with a 1 indicating a lower functional status of quality of life). Biochemical data of hemoglobin, albumin, and fasting glucose were obtained.

### Scintigraphy

Any medications that could alter the bowel motility such as opioids, anticholinergics, tricyclic antidepressants, calcium channel blockers, proton pump inhibitors, selective serotonin receptor inhibitors, macrolides, and dopamine antagonists were paused 3 days before scintigraphy. On the day of the scan, patients were only allowed to drink water for the last 12 h. Radiation-emitting metal clips (position markers) were attached to the right lateral costal arch and left lateral anterior superior iliac spine. The patients received an intravenous injection of 150 MBq 99mTc Bromo-biliaron (99mTc, i.e., technetium) to mark the bile acids. Ten minutes later, they ingested a standardized omelet containing two eggs, two tablespoons of flour, salt, 1 dl of 3% fat milk, 5 g of margarine, and 15 MBq 111In-DTPA (111In, i.e., indium) to mark the food. The first scan was done after an additional 10 min, i.e., 20 min after the administration of 99mTc.

### Data Collection

Data was collected with a gamma camera with an attached computer tomography (CT) scan. A series of 1-min anterior and posterior planar acquisitions were made every 10 min in a total of 180 min. Two energy windows were used to separate the information gathered from each of the two isotopes: 99mTc (126.45–154.55 keV) and 111In (157.32–184.68 keV). At the end of the scintigraphy, the patient performed a conventional low-dose CT scan and a plain-film X-ray of the abdomen.

### Analysis

A new protocol for the analysis of the scintigraphy was developed in-house and tested. Images were corrected for radiation decay and corrected for radiation spillover from 111In to the 99mTc acquisition energy window (down scatter). The calculated fraction was estimated to be 0.4 based on phantom measurements.

### Image Analysis

**Fig. 1:**
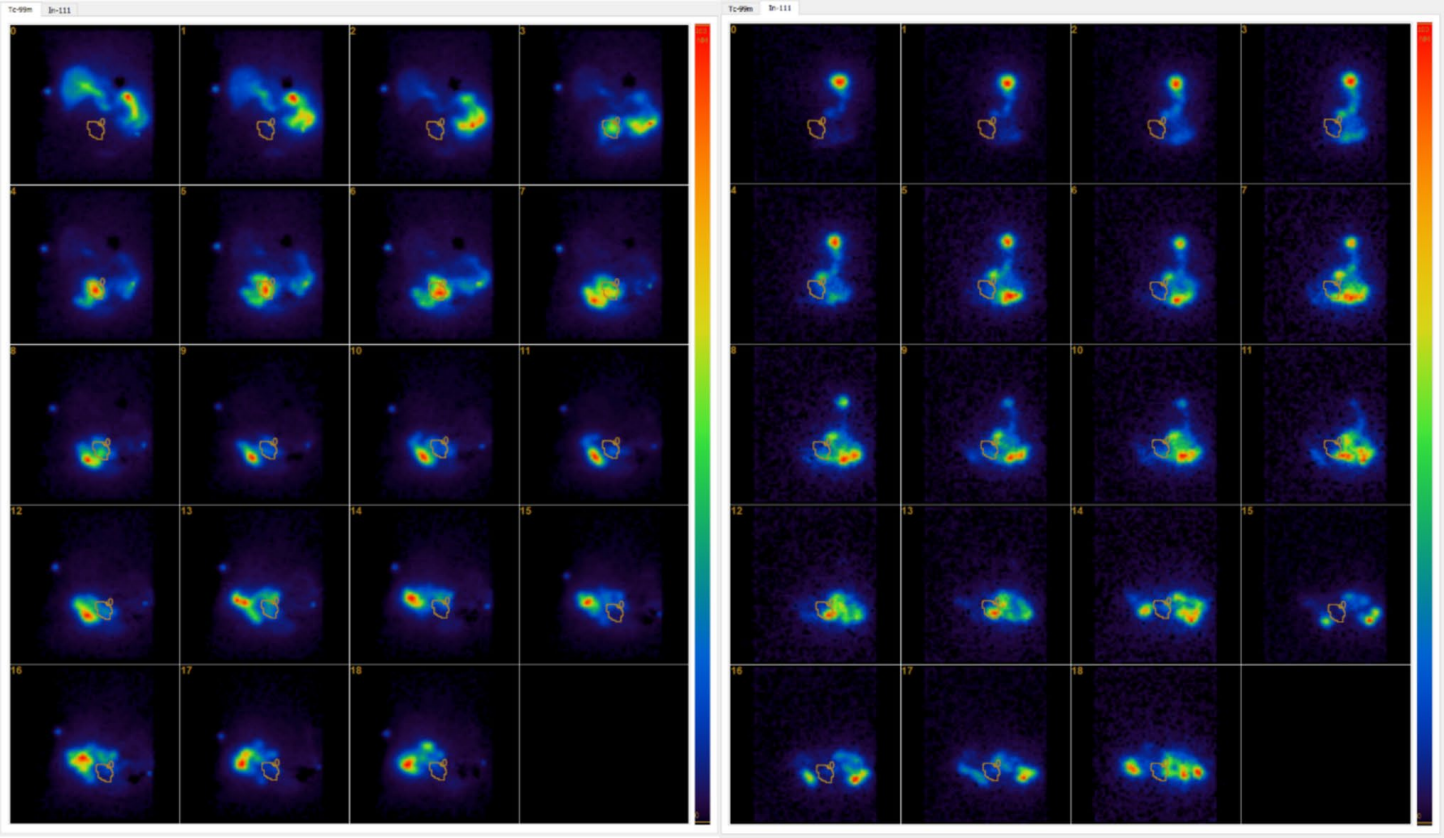
Typical scintigraphy of bile and food transit to the enteroenterostomy (EE). The EE has been drawn with a yellow circle. The color indicates the radioactive uptake ranging from dark blue (low uptake) to red (high uptake). Tc-99 m = 99Tc Bromo-biliaron (bile). In-111 = 111In-DTPA (food)

**Fig. 2:**
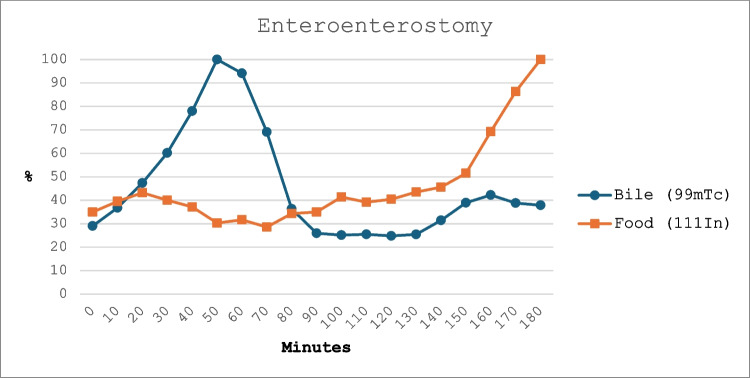
Scintigraphic uptake of bile and food at the enteroenterostomy (EE). Study duration is shown on the *X*-axis, with images acquired every 10 min. Percent (%) of maximal radioactive uptake at the EE is shown on the *Y*-axis. The blue line represents bile (99mTc), and the orange line represents food (111In)

The CT scan, abdominal X-ray, and the visual scintigraphy picture were used together to assess the location of landmarks such as the gastric tube, liver, duodenum, and enteroenterostomy. Region(s) of interest (ROI) was individually marked on the 99mTc images for the gastric tube and enteroenterostomy and triangulated to the position markers. Every ROI was analyzed individually and imported onto the 111In images to get the corresponding location for the food uptake. The time activity changes in the specified ROIs were visualized for each isotope with a line plot containing time stamps on the *X*-axis and % of maximal uptake on the *Y*-axis (Figs. 1 and 2).

#### Gastric Emptying

Gastric emptying was calculated according to international guidelines [16, 17] and presented primarily as the gastric emptying half-time as described by others [18, 19].

#### Enteroenterostomy

To correct for bowels outside of the EE, emitting radiation and interfering with the ROI uptake, and help with the anatomical triangulation, a few assumptions were made to aid the analysis: (1) if the line plot is biphasic, the first peak is assumed to be proximal bowel interference, and (2) if the radioactive uptake at the initial image is under 20% of maximal uptake, the uptake threshold for the ROI is 50%. If the uptake at the initial image is over 20%, the uptake threshold for the ROI is 60%. (3) A significant spike in uptake for both isotopes indicates an area where both food and bile transit. Gastrointestinal transit times to the EE were presented as follows: (1) food or bile time to EE, defined as the time point when the uptake starts to exceed the predefined threshold at the ROI; (2) food or bile time at EE, defined as the duration of uptake exceeding the threshold at the ROI; and (3) mixing at EE, defined as the duration of uptake exceeding the threshold at the ROI for both food and bile. The patients were stratified into two groups based on the definition of diarrhea: those with ≥ 3 stools per day and those with < 3 stools per day [20].

### Statistical Analysis

Parametric data were presented as mean and standard deviation (SD), and non-parametric data were presented as median and interquartile range (IQR). Patients with a transit time over 180 min were right-censored and analyzed as having a transit time of 180 min. Mann–Whitney *U* test was used for comparisons between groups for continuous non-parametric data. A *p* value of 0.05 was considered significant. Excel® was used for charts and descriptive statistics and SPSS® for the statistical analysis. The custom analysis program and detailed methodology are available from the corresponding author upon reasonable request.

## Results

Ten patients (five males) with a mean age of 52 ± 10 years were included. BMI was reduced from 53.0 ± 8.0 at surgery to 35.3 ± 6.9. On a scale of 1 to 5, with 5 being excellent, the mean general health was scored 3.1 ± 1.0. The FIQL scores, 3.2 to 3.6 across the four different domains, indicated reduced functional status of quality of life. Six patients had a stool frequency of ≥ 3 times per day and gases/flatus at least weekly. Four reported abdominal pain at least weekly. All but one patient adjusted their diet to mitigate these adverse symptoms. However, mean hemoglobin, albumin, and fasting glucose were within normal range. Table 1 shows demographics, FIQL, and biochemical profile.

**Table 1:** Demographics, FIQL, and biochemical profile

Demographics	
Gender, male, *n* (%)	5 (50)
Age at study, mean ± SD	52 ± 10
BMI at surgery, kg/m^2^, mean ± SD	53.0 ± 8.0
BMI at study, kg/m^2^, mean ± SD	35.3 ± 6.9
Diabetes, *n* (%)	0 (0)
Hypertension, *n* (%)	4 (40)
Hyperlipidemia, *n* (%)	0 (0)
Cardiovascular disease, *n* (%)	2 (20)
Reflux/ulcer, *n* (%)	2 (20)
Depression, *n* (%)	1 (10)
Quality of life	
General health, mean ± SD^a^	3.1 ± 1.0
FIQL lifestyle, mean ± SD^a^	3.3 ± 0.9
FIQL coping/behavior, mean ± SD^a^	3.2 ± 0.8
FIQL depression/self-perception, mean ± SD^a^	3.4 ± 0.8
FIQL embarrassment, mean ± SD^a^	3.6 ± 0.8
Bowel symptoms	
Stool frequency > 3 times per day, *n* (%)	6 (60)
Abdominal pain, a few times per week or more, *n* (%)	4 (40)
Gases/flatus, a few times per week or more, *n* (%)	6 (60)
Alteration of diet, *n* (%)	9 (90)
Laboratory values	
Hemoglobin, g/L, mean ± SD	133.8 ± 11.5
Albumin, g/L, mean ± SD	36.2 ± 3.3
Fasting glucose, mmol/L, mean ± SD	5.2 ± 0.8

*SD* standard deviation, *BMI* body mass index, *FIQL* fecal quality of life scale

^a^Scale ranging from 1 to 5, with 1 indicating lower functional status and quality of life

### Gastric Emptying

**Fig. 3:**
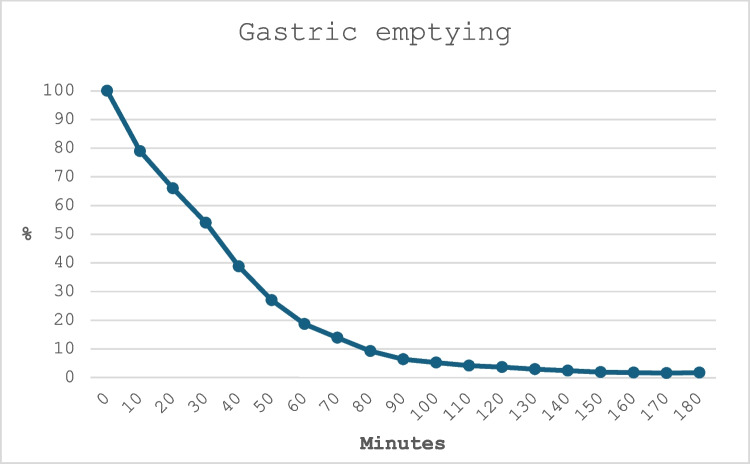
Gastric emptying median for all study patients. Study duration is shown on the *X*-axis, with images acquired every 10 min. Median percent (%) of maximal radioactive uptake at the stomach is shown on the *Y*-axis. Blue line represents bile food (111In)

Gastric emptying half-time was 40 min (IQR 30–42.5). At 1 h, median remaining uptake was 18% (IQR 11–30). Rapid gastric emptying, defined as less than 30% uptake at 1 h in a normal reference population [17], was seen in nine (90%) of the study patients. Figure 3 shows the gastric emptying median across all study patients.

### Enteroenterostomy and Mixing of Bile and Food in the Common Channel

All scintigraphic examinations were successfully performed and gave interpretable data. Gastrointestinal transit times to the EE, indicating the start of the common channel, showed great variability both between study participants as well as between bile and food, as shown in Table 2. Transit time to the EE was 70 min (IQR 55–138) for food and 105 min (IQR 38–148) for bile. The duration of significant amounts of bile and food at the EE was 80 min (IQR 43–125) min for food and 60 min (IQR 33–83) for bile. Six of ten patients (60%) showed any simultaneous presence of bile and food at the EE; in those, the overlap lasted for a short duration of 40 min (IQR 8–73). A typical scintigraphy is demonstrated in Figs. 1 and 2.

**Table 2:** Transit of food and bile to the enteroenterostomy

	Transit times				Difference	Mixing
Study patient	Food time to EE^1^	Bile time to EE^2^	Food time at EE^3^	Bile time at EE^4^	Time to EE difference^5^	Mixing at EE^6^
1	70	130	110	40	60	40
2	70	30	50	70	40	30
3	110	170	50	10	60	0
4	40	90	140	90	50	50
5	60	40	120	140	20	120
6	160	30	20	50	130	0
7	60	140	120	40	80	40
8	130	> 180	50	-	> 40	-
9	30	100	150	80	70	80
10	> 180	110	-	70	> 70	-
Median (IQR)	70 (55–138)	105 (38–148)	80 (43–125)	60 (33–83)	60 (40–73)	40 (8–73)

*EE* enteroenterostomy, *IQR* interquartile range

^1^Time point when food reaches EE (min)

^2^Time point when bile reaches EE (min)

^3^Duration of food at EE (min)

^4^Duration of bile at EE (min)

^5^Bile and food transit time difference to EE (min)

^6^Simultaneous presence of food and bile at EE (min)

### Transit Times and Symptoms

The patients were divided into two groups based on stool frequency. In the group with stool frequency ≥ 3 times per day, food (70 min) reached the EE faster than bile (115 min), while in the group with < 3 stools/day, bile (110 min) reached the EE faster than food (75 min). There was no statistically significant difference between the groups in time to EE for food, nor time to EE for bile (*p* = 0.453 and 0.521, respectively).

## Discussion

This is the first study exploring the transit times of food and bile simultaneously after BPD/DS surgery with a dual-isotope scintigraphy. The successful marking of bile acids and food with 99mTc and 111In, respectively, provided alimentary and biliary transit times in all patients. Gastric emptying was fast. Median time to EE was 70 min for food and 105 min for bile; however, a great variability both between study participants as well as between bile and food was observed.

The characteristics of the included patients with equal proportion of men and women in their forties, having had a weight loss of 17.7 BMI units, are typical for BPD/DS. To avoid difficulties with altered biliary anatomy, patients having had cholecystectomy were excluded. As there is no bariatric quality of life instrument considering gastrointestinal adverse symptoms sufficiently, we used the validated FIQL tool. However, because FIQL is aimed at patients with fecal incontinence, it is suboptimal for assessment of postoperative gastrointestinal symptoms in an MBS population such as malabsorption and dumping. FIQL is designed with four separate domains and lacks a composite score which impairs comparisons between patients or groups. Our patients reported a clear impact on quality of life from gastrointestinal adverse symptoms, with FIQL domain scores between 3.2 and 3.6 out of 5. Of note, this was similar to patients with anal incontinence, awaiting elective gastrointestinal surgery [21] or seeking bariatric surgery [22].

The study duration of 180 min was too short to measure the transit times to the EE in all patients, with 3 values being right-censored at 180 min. This might both underestimate transit times and differences between the groups, and future studies should increase the scanning duration. There was no statistical difference in transit times between our groups; however, we might miss potential associations due to being underpowered. In a study that used a motility capsule, the total small bowel transit time in BPD/DS patients was estimated to be 2.8 ± 2.0 h [23]. However, transit times to the EE have been notoriously difficult to measure. The total small bowel length in a non-operated population ranges from 400 to over 1000 cm [24–26]. The included patients had an alimentary limb of 150 cm and a common channel of 100 cm; thus, the length of the biliary limb varied substantially and was also assumed to exceed the alimentary limb in length. When comparing a standard BPD/DS with a long alimentary limb duodenal switch (LADS), the latter resulted in significantly fewer complaints for abdominal bloating and stool odors, however, at the cost of lower weight loss [27]. Many studies have tried different lengths of the common channel to combat complications related to the malabsorptive part of the BPD/DS, but few studies include details of gastrointestinal symptoms [28, 29]. Thus, to what extent the variation in transit times and time for mixing of bile acids and food in the common channel can explain the severity of gastrointestinal adverse symptoms is uncertain.

As expected, the present gastric emptying was faster compared to a non-operated population [16]. This is probably explained by postoperative alteration in gastric anatomy and physiology. For example, the normal inhibition of gastric motility, induced by duodenal distension, is lacking after the exclusion of passage of food into the duodenum and the divided vagal branches when mobilizing the pyloric area [30]. In a former study by our group on BPD/DS patients [19], the half-emptying time was 28 ± 16 min compared to 40 min (IQR 30–42.5) in this study. This difference can be explained by a significantly smaller 111In-labelled omelet, composed of ½ egg compared to two eggs in this study. In patients after sleeve gastrectomy, rapid gastric emptying has been associated to better weight loss [31]. Unfortunately, our small sample size prevented us from studying this.

### Limitations and Potential Improvements

Being the first study exploring the transit times of bile and food with a dual-isotope scintigraphy, several limitations are due to the small sample and the multi-step approach needed to calculate the results. Comparisons between groups were therefore based on symptomatology and not objective markers such as fecal fat quantification, fecal elastase, or 75-selenium homocholic acid taurine (SeHCAT) test for bile acid malabsorption. The methodological challenge in defining anatomical landmarks, especially the enteroenterostomy, is a considerable limitation. The enteroenterostomy can potentially move with normal bowel peristalsis and might also be affected by positional changes during the examination. Due to technical reasons, we had to perform the scintigraphy in a sitting position, while the CT scan and X-ray were done in a supine position. Radiopaque endoscopic markings at the enteroenterostomy would have solved this problem; however, these are not easy to get in place. Furthermore, interference from bowel segments not related to the ROI poses a challenge when interpreting the raw data. Since established alternatives were lacking, a novel program had to be created in-house which may affect reproducibility. The multiple steps needed to calculate the results as well as the use of non-validated radioactive uptake threshold levels provide an inherent margin of error. Lastly, we did not have a control group, so differences from normal physiology were derived from published values. We recommend future studies to include more patients, as well as different control groups, and use a bariatric-specific gastrointestinal quality of life questionnaire.

## Conclusion

Dual-isotope scintigraphy enables assessment of gastric and small bowel transit after BPD/DS, with some limitations. Although transit times were variable, we believe that the novel imaging technique can add valuable information in patients with severe gastrointestinal symptoms.

## Data Availability

No datasets were generated or analysed during the current study.
